# Effects of baked and raw salmon fillet on lipids and n-3 PUFAs in serum and tissues in Zucker fa/fa rats​​​​​​​​​​​​​​​​​​​​

**DOI:** 10.1080/16546628.2017.1333395

**Published:** 2017-06-12

**Authors:** Linn A. Vikøren, Aslaug Drotningsvik, Marthe T. Bergseth, Svein A. Mjøs, Nazanin Mola, Sabine Leh, Gunnar Mellgren, Oddrun A. Gudbrandsen

**Affiliations:** ^a^ Dietary Protein Research Group, Department of Clinical Medicine, University of Bergen, Bergen, Norway; ^b^ Department of Clinical Science, University of Bergen, Haukeland University Hospital, Bergen, Norway; ^c^ Department of Chemistry, University of Bergen, Bergen, Norway; ^d^ Nofima BioLab, Bergen, Norway​​​​​; ^e^ Department of Pathology, Haukeland University Hospital, Bergen, Norway; ^f^ Department of Clinical Medicine, University of Bergen, Bergen, Norway; ^g^ Department of Clinical Science, KG Jebsen Center for Diabetes Research, University of Bergen, Haukeland University Hospital, 5021Bergen, Norway; ^h^ Hormone Laboratory, Haukeland University Hospital, 5021Bergen, Norway

**Keywords:** Fish intake, fish, preparation methods, cholesterol, triacylglycerol

## Abstract

Knowledge of the health impact of consuming heat-treated versus raw fish fillet is limited. To investigate effects of baked or raw salmon fillet intake on lipids and n-3 PUFAs in serum and tissues, obese Zucker fa/fa rats were fed diets containing 25% of protein from baked or raw salmon fillet and 75% of protein from casein, or casein as the sole protein source (control group) for four weeks. Salmon diets had similar composition of amino and fatty acids. Growth and energy intake were similar in all groups. Amounts of lipids and n-3 PUFAs in serum, liver and skeletal muscle were similar between rats fed baked or raw salmon fillet. When compared to the control group, rats fed baked salmon had lower serum total and LDL cholesterol and higher serum triacylglycerol levels. Both raw and baked salmon groups had lower HDL cholesterol level when compared to control rats. In conclusion, baking as a preparation method does not alter protein and fat qualities of salmon fillets, and intake of baked and raw salmon fillets gave similar effects on lipids and n-3 PUFAs in serum and tissues from rats.

## Introduction

Obesity is a major public health problem. Fish consumption has been associated with cardio protective properties [1,2], and may therefore be a possible preventive and therapeutic approach for combating metabolic disturbances such as dyslipidemia, related with overweight and obesity. Yet, reports on associations of fish consumption and reduced cardiovascular disease (CVD) risk are inconsistent [3,4]. Conflicting associations of fish consumption and risk of CVD have been suggested to be related to differences in preparation methods and type of fish consumed in different population groups [5,6]. Hitherto, few studies have directly assessed effects of differently prepared fish fillet on lipid metabolism and CVD risk factors.

Health benefits of fish consumption have traditionally been attributed to the long chain n-3 PUFAs eicosapentaenoic acid (EPA) and docosahexaenoic acid (DHA), which are plentiful in fillet from fatty fish [7]. Fish is also a good source of several different nutrients, including high-quality proteins and vitamin D. Fish proteins are now being recognized for their potential beneficial effects on lipid metabolism and CVD risk factors [8].

Health effects of fish intake may be affected by preparation methods, as heat treatment can affect nutrient quality of proteins, fat and micronutrients, the use of fat or oil during frying may increase the content of unfavourable fatty acids, and boiling/blanching of fish may lead to loss of both water-soluble proteins and fat from the fish [9–11]. Advanced glycated end products (AGEs) are created in spontaneous reactions, such as between reducing sugars and free amino groups. AGEs are highly oxidative and may cause oxidative stress, thus creating an environment of inflammation [12]. AGEs are present in uncooked animal-derived foods and additional AGEs are generated during heat processing. Temperature and duration of thermal preparation of foods are essential for the amount of AGEs generated [13,14]. Carboxymethyl-lysine (CML) and methylglyoxal (MG) are the best characterized AGEs and are commonly measured when assessing AGEs in diet and as an indicator of AGE-burden in the body [12–14]. Diets high in AGEs have been reported to increase circulating levels of CML [15].

Boiling, frying (not deep-frying) and baking/roasting are the most common preparation methods of fish in Norway [16]. Baking is a more gentle preparation method than frying and deep-frying, as temperatures are lower, and additional oils or fats are not needed. Eating raw fish is becoming increasingly popular in many countries including Norway, and consumers perceive sushi as a healthy food choice [17,18]. Still, little is known about the nutritional value of heat-treated fish versus raw fish in the human diet, and whether different preparation methods could affect the health benefits of eating fish.

The Zucker fa/fa rat is a well characterized and widely used model for human obesity. The rats develop obesity a few weeks after birth and develop obesity-related comorbidities such as dyslipidemia and fatty liver [19]. Previous studies from our lab with diets containing 25% of protein from fish show that proteins from different fish species, i.e. cod, herring and salmon, affect growth, glucose regulation and lipid metabolism in obese Zucker fa/fa rats [20,21]. In the present study we investigated the effects of diets containing the same amount of protein from baked or raw salmon fillet. A diet with 25% of protein from fish is believed to be more comparable to the amount of fish protein consumed by humans, in contrast to experimental diets with 50 or 100% of proteins from fish. Using salmon fillet as a source of proteins provides a different approach than consumption of purified hydrolysed protein or fish oil alone, and might be more transferable to human consumption of fish, as fish is normally consumed as fillet prepared by different methods and as part of a varied diet. The aim of the present exploratory study was to investigate whether baking of salmon fillet affected the composition of fatty acids and amino acids in the fillets, and to compare the effects of baked and raw salmon on lipids and n-3 PUFAs in serum, liver, skeletal muscle and epididymal white adipose tissue (WATepi) in Zucker fa/fa rats.

## Materials and methods

### Ethical statement

The study protocol was approved by the National Animal Research Authority of Norway, in accordance with the Animal Welfare Act and the regulation of animal experiments (approval no 2014/6979). The animal care and use programme at the Faculty of Medicine and Dentistry at University of Bergen is accredited by the Association for Assessment and Accreditation of Laboratory Animal Care International.

### Design

Eighteen male Zucker fa/fa rats (HsdHlr:ZUCKER-Leprfa) obtained from Harlan Laboratories (Indiana, USA) were randomized into three weight-matched dietary intervention groups. The rats were housed in pairs in Makrolon IV cages (EHRET GmbH & Co.), under standard conditions with a constant humidity of 65 ± 15%, temperature of 20 ± 3⁰C and a light–dark cycle of 12 hours. Rats were acclimatized under these conditions for at least one week before the start of the experiment. Intervention was initiated when rats weighed 350 ± 20 g. Rats were weighed weekly during the experimental period. Rats were fed the intervention diets for four weeks and had ad libitum access to feed and tap water. Freshly thawed feed was provided daily in ceramic bowls, and due to powder formula feed the rats were given wooden chewing sticks. One week before euthanasia, rats were housed individually for 24 hours on a grid in standard cages to evaluate feed intake and faeces output.

### Diets

Semi-purified diets were prepared according to the AIN-93G recommendations and contained 20 wt% proteins (Table 1) [22]. In the salmon diets, baked or raw salmon fillet constituted 25% of the protein, and casein was added to provide 75% of the protein. Casein served as the sole protein source in the control diet.

Casein was purchased from Sigma-Aldrich (Munich, Germany). Skin-free salmon fillets from Atlantic Salmon (farmed Salmo salar, fed 6% EPA+DHA of fatty acids until 1200 g bodyweight and 4.5% EPA+DHA in following feeds [23]) were provided by Leroy Seafood Group (Hordaland, Norway). All other dietary ingredients were purchased from Dyets Inc. (Pennsylvania, USA). Baked fish filets were prepared in oven at 180⁰C for 20 minutes, no oil or fat were added when baking the fish. Baked and raw salmon fillets were minced, freeze dried and grinded before being mixed with the other ingredients in the diets.

### Analysis of diets and salmon fillet

The contents of energy, total fat, total amino acids, free amino acids and taurine in the feeds, as well as vitamin D3 content of the salmon fillets were analysed by Nofima, BioLab (Hordaland, Norway). Cholesterol in the diets was extracted according to the method described by Bligh and Dyer [24], using a mixture of methanol and chloroform. The extracted lipid fraction was evaporated to dryness with nitrogen and re-dissolved in isopropanol before cholesterol was analysed on the Cobas c 111 system (Roche Diagnostics GmbH, Marburg, Germany) using the CHOL2 (Cholesterol Gen.2) kit from Roche.

### Euthanasia, sampling and measurements

After a 12 h overnight fast with access to water, rats were anaesthetized with Isoflurane (Isoba vet, Intervet, Schering-Plough Animal Health, Boxmeer, the Netherlands) and a mixture of nitrous oxygen (N_2_O) and oxygen. Blood was drawn from the heart and collected in Vacuette Z Serum Clot Activator Tubes for isolation of serum (Greiner Bio One, Austria). Liver, thigh skeletal muscle and WATepi were harvested and weighed. Serum and organs were immediately frozen in liquid nitrogen and stored at −80°C until further analyses. The rats were weighed before fasting. Body length of the rats was measured with a ruler.

### Analyses in serum

Total cholesterol, low density lipoprotein (LDL) cholesterol, high density lipoprotein (HDL)​​​​​ cholesterol, triacylglycerol (TAG) and total bile acids in fasting serum samples were analysed at the Laboratory of Clinical Biochemistry at Haukeland University Hospital (Bergen, Norway) by accredited methods. Serum NEFA and free cholesterol were analysed on the Cobas c 111 system (Roche) using the NEFA FS kit (DiaSys Diagnostics Systems GmbH, Holzheim, Germany) and the free cholesterol FS kit (Diagnostic Systems). The cholesteryl ester concentration was calculated as the difference between total and free cholesterol. Serum vitamin D was measured by ELISA, using the 25-OH Vitamin D total (Rat) kit (EIA-5532, from DRG Instruments GmbH, Germany). Serum CML and MG concentrations were measured using ELISA-kits from OxiSelect™ (STA-816 and STA-811, Cell Biolabs Inc., San Diego, USA).

### Analyses of lipids in liver, skeletal muscle and faeces

Lipids in liver, skeletal muscle and faeces were extracted according to the method described by Bligh and Dyer [24], using a mixture of methanol and chloroform. The extracted lipid fraction was evaporated to dryness with nitrogen and re-dissolved in isopropanol before TAG and total cholesterol were analysed on the Cobas c 111 system using the TRIGL (Triglycerides) and CHOL2 (Cholesterol Gen.2) kits from Roche. Liver slices (approximately 3 mm thick) were fixed in 4% buffered formaldehyde, processed by standard procedures and embedded in paraffin. Three micrometer sections were stained with hematoxylin-eosin. Slides were scanned with ScanScope® XT (Aperio) at ×40 resulting in a resolution of 0.25 micrometer per pixel. Virtual slides were viewed in ImageScope 12. Fat storage in liver cells was quantified by automatic image analysis using the Aperio deconvolution algorithm. The fat content was calculated as a percentage of liver by using the total stained area (mm^2^) and total analysis area (mm^2^).

### Analyses of bile acids in faeces

Total faecal bile acids (3α-hydroxy bile acids) were measured in freeze-dried faeces by the method described by Suckling et al. [25], using Chromabond C18 ec (3 ml/200 mg, Macherey-Nagel, Düren, Germany) and the enzymatic bile acid kit from Diazyme Laboratories, Inc. (California, USA) on the Cobas c111 system.

### Analyses of fatty acid composition in diets, serum, liver, skeletal muscle and white adipose tissue

Lipids were extracted from the different diets, liver and skeletal muscle as described above. The extracts were methylated and extracted twice with isooctane as previously described [26]. Heneicosanoic acid was added as an internal standard. Serum and WATepi samples were methylated without prior lipid extraction. Methyl esters were quantified by an Agilent 7890 gas chromatograph equipped with flame ionization detector and a BPX-70 capillary column as described in [27]. The fatty acid standards were identified by gas chromatography-mass spectrometry (GC-MS) using the BPX-70 column and methodology as previously described [28].

### Aim and sample size calculation

The aim of this study was to investigate whether baking of salmon fillet would affect the composition of fatty acids and amino acids in the fillet when compared to raw salmon fillet, and to compare the effects of intake of diets containing baked or raw salmon on lipids and n-3 PUFAs in serum and tissues in Zucker fa/fa rats. The sample size calculation for the present study was conducted using unpublished data from a pilot study with dietary fish oil in Wistar rats, and the adequate sample size was calculated to be four–five rats in each group (depending on the variables analysed) with a power of 80% and alpha = 0.05.

### Statistical analyses

Data were assessed for normal distribution by the Shapiro-Wilks test and Q-Q plots. Most variables were normally distributed.​​​​​ All variables were compared between groups using one-way analysis of variance (ANOVA) and the Tukey HSD post-hoc test, when appropriate. Non-normally distributed data were simultaneously assessed using the non-parametric Kruskal–Wallis test method; however, findings were similar and for simplicity only results from ANOVA testing are presented in the article. The level of statistical significance was set at p < 0.05. Statistical analyses were performed using IBM SPSS Statistics 23 (IBM Corporation, New York, USA). Data are presented as mean with standard deviations. One rat in the control group had to be euthanized in the first week of the study due to a serious lesion and poor health and is not included in the results, therefore n = 5 in the control group and n = 6 in each of the salmon groups.

## Results

### Diets

The compositions of the baked and raw salmon diets were comparable, with similar compositions of amino acids and fatty acids (Table 2). The energy contents of salmon diets were comparable with 18.9 kJ/g feed in the baked salmon diet and 19.1 kJ/g feed in the raw salmon diet. The control diet had slightly lower energy content than salmon diets with 18.1 kJ/g feed. Amino acid composition was generally comparable between salmon diets and control diet, however taurine was present only in the salmon diets (Table 2). Contents of free amino acids in the diets were below limit of detection except for methionine which was added in its free form to the diets (data not presented). Ratios of lysine/arginine and methionine/glycine were marginally lower in both salmon diets compared to the control diet. The cholesterol content was low in all diets; 0.45, 0.55 and 0.25 µmol cholesterol per gram diet in the baked salmon, raw salmon and control diet, respectively. The fatty acid compositions of the salmon diets were markedly different from that of the control diet, with higher amounts of 16:0, 18:0, 18:1 n-9, 18:1 n-7 and alpha-linolenic acid (ALA, 18:3 n-3) in the former diets (Table 2). The long chain n-3 PUFAs EPA (20:5 n-3), docosapentaenoic acid (DPA, 22:5 n-3) and DHA (22:6 n-3) were present in both salmon diets, but were not detected in the control diet. Levels of linoleic acid (18:2 n-6) were comparable between all three diets. Salmon fillet used in the salmon diets contained 4µg vitamin D3 per 100 g fillet.

**Table 1.** Composition of diets.​​​​​

g/kg	Baked salmon diet	Raw salmon diet	Control diet
Casein^a^	162.2	162.2	216.2
Freeze–dried baked salmon fillet^b^	100.0		
Freeze–dried raw salmon fillet^b^		100.0	
Soya oil	70.0	70.0	70.0
Sucrose	90.0	90.0	90.0
Cornstarch	466.0	466.0	512.0
Cellulose	50.0	50.0	50.0
Mineral mix (AIN-93-MX)	35.0	35.0	35.0
Vitamin mix (AIN-93-VX)	10.0	10.0	10.0
L-cysteine	3.0	3.0	3.0
L-methionine	1.6	1.6	1.6
Choline bitartrate^c^	2.5	2.5	2.5
Tert-butylhydroquinone	0.014	0.014	0.014
Growth and maintenance supplement^d^	10.0	10.0	10.0

^a^Contains 92.5% crude protein.

^b^Contains 50% crude protein.

^c^Contains 41.1 % choline.

^d^Contains 2.5 g vitamin B_12_, 2.5 g vitamin K_1_ and 995 g sucrose per kg.

**Table 2.** Contents of indispensable amino acids, the functional amino acid glycine, the conditionally essential amino acid arginine, taurine, ratios of lysine/arginine and methionine/glycine and fatty acids* in the diets (g/kg diet).

g/kg diet	Baked salmon diet	Raw salmon diet	Control diet
*Amino acids*			
Arginine^†^	7.9	7.5	6.4
Glycine	5.0	4.8	3.6
Histidine	5.6	5.3	5.1
Isoleucine	10.0	9.4	9.2
Leucine	17.0	17.0	16.0
Lysine	16.0	15.0	14.0
Methionine	7.6	7.1	6.4
Phenylalanine	9.7	9.1	9.1
Threonine	8.0	8.0	7.2
Valine	13.0	12.0	12.0
Taurine	0.1	0.1	ND
Lys/Arg ratio	2.0	2.0	2.2
Met/Gly ratio	1.5	1.5	1.8
*Fatty acids*			
16:0	9.6	9.6	7.5
18:0	3.0	3.0	2.5
18:1 n-9	24.8	26.1	13.9
18:1 n-7	1.6	1.7	0.9
18:2 n-6	34.5	33.9	32.7
18:3 n-3	5.0	5.1	3.9
20:5 n-3	0.5	0.6	ND
22:5 n-3	0.2	0.2	ND
22:6 n-3	1.0	1.1	ND

ND, not detected.

*Only fatty acids found in levels > 0.1 g/kg diet are shown.

†Arginine can be synthesized by the rat, but not in sufficient amounts to meet the demands for normal growth.

### Energy intake, growth, body and organ weights

Body weight at baseline, body weight at endpoint, growth, ratio of weight/square body length, as well as weights of liver, skeletal muscle (thigh) and WATepi relative to body weight were similar between baked salmon, raw salmon and control groups (Table 3). Energy intake and dry faecal output per kilo body weight were similar in all three groups.

**Table 3.** Body weight at baseline, growth and organ weights relative to body weight at time of euthanasia, energy intake and faecal output one week before euthanasia.

	Baked salmon diet		Raw salmon diet		Control diet		ANOVA
	Mean	SD	Mean	SD	Mean	SD	P
Body weight at baseline (g)	356	13	356	14	357	14	0.98
Body weight at endpoint (g)	541	23	553	27	570	21	0.17
Growth (%)	52	3	48	12	45	11	0.50
Body weight/square body length (without tail) ratio (kg/m^2^)	10.1	0.3	9.9	0.4	10.0	0.5	0.78
Liver (g/kg body weight)	36.5	4.8	36.6	3.7	42.9	6.3	0.086
Skeletal muscle (g/kg body weight)	2.9	0.5	3.0	0.5	3.0	0.4	0.83
Epididymal white adipose tissue (g/kg body weight)	31.8	4.6	29.6	4.7	31.3	5.9	0.75
Energy intake (kJ/kg body weight)	1056	142	1194	121	1200	119	0.14
Faecal output (g dry faeces/kg body weight)	3.9	2.0	5.0	1.8	5.9	0.9	0.19

Values are mean and standard deviations, n = 5 in control group, n = 6 in baked salmon group and n = 6 in raw salmon group.

No significant differences were found between groups (p < 0.05; evaluated by one-way ANOVA).

### Serum lipids, bile acids, vitamin D, MG and CML

Serum concentrations of lipids, bile acids, vitamin D, NEFA, MG and CML were similar between rats fed baked or raw salmon diets (Table 4). Circulating concentrations of NEFA, vitamin D, MG and CML were also similar between salmon groups and control group. Serum concentrations of total cholesterol, HDL cholesterol, LDL cholesterol and bile acids were significantly lower, and that of TAG was significantly higher in the baked salmon group compared to the control group. In the raw salmon group, the serum concentration of HDL cholesterol was significantly lower, and there were tendencies towards lower LDL cholesterol and higher TAG in the raw salmon group compared to control (p values of 0.055 and 0.067, respectively).

**Table 4.** Concentrations of serum lipids, bile acids, vitamin D, MG-BSA and CML-BSA.

	Baked salmon diet		Raw salmon diet		Control diet		ANOVA	Post hoc test		
	Mean	SD	Mean	SD	Mean	SD	P	P*	P‡	P†
Total cholesterol (mmol/l)	4.9	0.8	5.4	0.8	6.3	0.6	0.022	0.53	0.018	0.13
HDL cholesterol (mmol/l)	2.7	0.2	3.3	1.3	5.3	0.3	0.00039	0.50	0.00039	0.003
LDL cholesterol (mmol/l)	0.9	0.3	1.1	0.4	1.5	0.3	0.0080	0.50	0.0067	0.055
Cholesteryl esters (mmol/l)	3.7	0.5	4.3	1.0	4.9	0.5	0.051			
TAG (mmol/l)	4.4	1.5	3.9	0.8	2.3	0.5	0.016	0.68	0.015	0.067
NEFA (mmol/l)	0.8	0.1	0.9	0.1	0.9	0.1	0.60			
Bile acids (µmol/l)	15.7	7.0	25.5	17.2	46.0	20.9	0.021	0.54	0.018	0.12
Vitamin D (ng/ml)	28.3	5.3	26.2	7.2	27.4	6.7	0.86			
MG-BSA^1^ (µg/ml)	4.9	0.6	5.0	0.7	4.4	1.3	0.56			
CML-BSA^2^ (µg/ml)	2.5	0.7	2.5	0.4	2.7	0.8	0.89			

Values are mean and standard deviations, n = 5 in control group, n = 6 in baked salmon group and n = 6 in raw salmon group.

^1^Methylglyoxal​​​​​

^2^Carboxymethyl-lysine

* Baked salmon vs raw salmon.

‡ Baked salmon vs control.

† Raw salmon vs control.

(p < 0.05 were considered significant; evaluated by one-way ANOVA and Tukey HSD post-hoc test).​​​​​

### Analyses in liver, muscle and faeces

**Figure 1.**
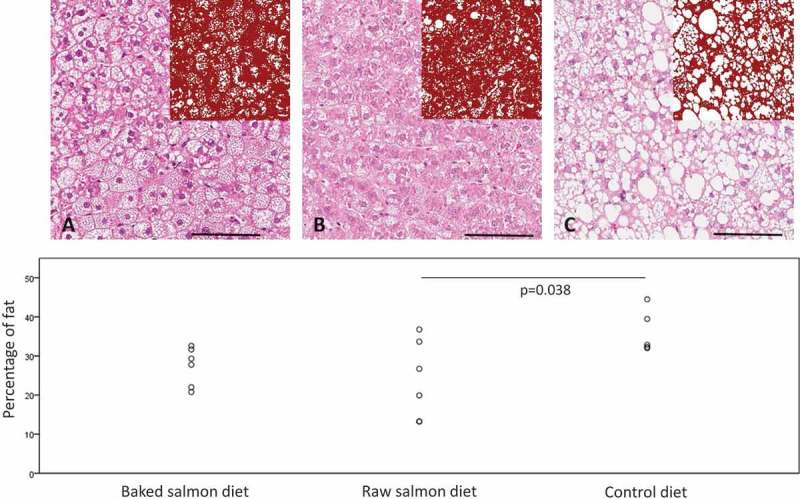
Representative images from liver sections showing steatosis in the groups fed with baked salmon diet (A) and raw salmon diet (B) compared to steatosis in the control group (C) (stain hematoxylin-eosin, scale bars 100 micrometer). Insets in the upper right corner display marked-up images for image analysis. The graph shows percentage fat content calculated by image analysis for individual rats.

Amounts of total cholesterol and TAG in liver and muscle, well as daily faecal excretion of cholesterol, TAG and total bile acids were similar in all groups (Table 5). Image analysis of the stained liver sections revealed no differences in percentage of fat between rats fed baked salmon or raw salmon (p = 0.71, Figure 1). The liver fat content was significantly lower in rats fed raw salmon diet (p = 0.038) and showed a weak tendency to be lower in rats fed baked salmon (p = 0.15) when compared to the control group.

**Table 5.** Concentrations of lipids in liver and skeletal muscle (thigh), and lipids and bile acids in 24 h faecal output.

	Baked salmon diet		Raw salmon diet		Control diet		ANOVA
	Mean	SD	Mean	SD	Mean	SD	P
*Liver*							
Cholesterol (µmol/g)	7.1	2.3	8.1	1.1	9.3	2.1	0.20
TAG (µmol/g)	125.3	30.3	117.7	39.2	147.1	43.2	0.44
*Skeletal muscle*							
Cholesterol (µmol/g)	1.6	0.1	1.7	0.2	1.6	0.2	0.78
TAG (µmol/g)	17.4	4.5	26.4	15.2	15.7	5.3	0.19
*Faeces*							
Cholesterol(µmol/24 h)	21.0	4.2	23.0	6.8	18.0	5.6	0.37
TAG (µmol/24 h)	2.6	1.3	3.3	2.5	2.3	0.8	0.61
Bile acids (µmol/24 h)	9.1	1.9	10.5	2.3	11.8	2.7	0.20

Values are mean and standard deviations, n = 5 in control group, n = 6 in baked salmon group, and n = 6 in raw salmon group.

No significant differences were found between groups (p < 0.05; evaluated by one-way ANOVA).

### Contents of n-3 and n-6 PUFAs in serum, liver, skeletal muscle and white adipose tissue

The contents of the n-3 PUFAs ALA, EPA, DPA and DHA in serum, liver and skeletal muscle were similar between the rats fed baked salmon and raw salmon (Table 6). Amounts of ALA, DPA and total n-3 PUFA were significantly lower in WATepi from rats fed baked salmon compared to rats fed raw salmon, with similar levels of EPA and DHA between the groups. The contents of n-6 PUFA in serum, liver, skeletal muscle and WATepi were mostly similar between the baked salmon and raw salmon groups, with the exception of higher 18:3n-6 in serum and lower 18:2n-6 in WATepi from rats fed the baked salmon diet when compared to rats fed the raw salmon diet. No differences were seen between the salmon fed groups for n-3/n-6 PUFA ratio in serum, liver, skeletal muscle and WATepi.

**Table 6.** n-3 and n-6 PUFAs in serum, liver, skeletal muscle and epididymal white adipose tissue.

	Baked salmon diet		Raw salmon diet		Control diet		ANOVA	Post-hoc test		
g/100 g fatty acids	Mean	SD	Mean	SD	Mean	SD	P	P*	P‡	P†
*Serum*										
18:3 n-3 (ALA)	0.71	0.17	0.81	0.22	0.36	0.05	0.0021	0.54	0.015	0.0020
20:5 n-3 (EPA)	1.40	0.14	1.35	0.22	0.39	0.10	8.6x10^−8^	0.86	2.1x10^−7^	3.8x10^−7^
22:5 n-3 (DPA)	1.19	0.21	1.19	0.28	0.62	0.07	0.00090	1.00	0.0018	0.0020
22:6 n-3 (DHA)	4.55	0.50	4.80	0.57	2.73	0.13	5.3x10^−6^	0.61	3.4x10^−5^	8.0x10^−6^
n-3 PUFA	7.86	0.84	8.18	1.22	4.11	0.13	4.0x10^−6^	0.81	1.8x10^−5^	7.3x10^−6^
18:2 n-6	12.2	0.96	13.4	1.30	10.6	0.32	0.0012	0.13	0.039	0.00087
18:3n-6	0.33	0.04	0.27	0.03	0.32	0.05	0.048	0.047	0.82	0.17
20:3n-6	1.20	0.12	1.12	0.12	0.76	0.12	9.7x10^−5^	0.59	0.0010	0.0061
20:4n-6	19.9	3.26	21.1	4.17	31.1	1.84	1.2x10^−5^	0.80	0.00019	0.00056
22:4n-6	0.37	0.09	0.33	0.07	0.52	0.02	0.0016	0.60	0.010	0.0016
22:5n-6	0.16	0.04	0.15	0.03	0.51	0.04	9.4x10^−8^	0.96	3.5x10^−7^	2.6x10^−7^
n-6 PUFA	34.2	3.3	36.4	3.4	43.8	2.3	0.00039	0.45	3.7x10^−5^	0.0035
n-3/n-6 PUFA ratio	0.23	0.03	0.23	0.05	0.09	0.01	2.1x10^−5^	0.98	4.9 x10^−5^	6.5x10^−5^
*Liver*										
18:3 n-3 (ALA)	0.76	0.20	0.80	0.17	0.35	0.03	0.00062	0.90	0.0021	0.00093
20:5 n-3 (EPA)	0.33	0.14	0.34	0.10	0.05	0.01	0.00066	0.99	0.0016	0.0013
22:5 n-3 (DPA)	0.49	0.18	0.52	0.14	0.18	0.02	0.0019	0.91	0.0057	0.0027
22:6 n-3 (DHA)	2.25	0.74	2.38	0.79	0.94	0.24	0.0056	0.94	0.014	0.0076
n-3 PUFA	3.83	1.09	4.04	1.07	1.52	0.26	0.00090	0.92	0.0028	0.0014
18:2 n-6	9.24	0.99	9.65	1.31	7.08	0.63	0.0026	0.78	0.011	0.0030
18:3 n-6	0.20	0.02	0.17	0.02	0.19	0.02	0.16			
20:3 n-6	0.41	0.11	0.42	0.17	0.17	0.05	0.0077	1.00	0.015	0.013
20:4 n-6	3.84	1.62	3.86	1.54	3.20	0.83	0.69			
22:4 n-6	0.12	0.03	0.12	0.04	0.12	0.03	1.00			
22:5 n-6	0.06	0.02	0.06	0.03	0.15	0.06	0.0021	1.00	0.0042	0.0042
n-6 PUFA	13.9	1.8	14.3	2.5	10.9	1.0	0.026	0.93	0.058	0.030
n-3/n-6 PUFA ratio	0.27	0.05	0.28	0.03	0.14	0.01	9.9x10^−6^	0.91	3.7x10^−5^	2.0x10^−5^
*Skeletal muscle*										
18:3 n-3 (ALA)	0.92	0.15	1.04	0.24	0.71	0.15	0.028	0.50	0.17	0.023
20:5 n-3 (EPA)	0.28	0.06	0.25	0.06	0.12	0.02	0.00022	0.62	2.5x10^−5^	0.0013
22:5 n-3 (DPA)	1.80	0.31	1.64	0.58	1.86	0.33	0.69			
22:6 n-3 (DHA)	6.16	1.24	5.61	2.26	4.30	0.96	0.19			
n-3 PUFA	9.17	1.48	8.55	2.69	6.98	1.17	0.20			
18:2 n-6	16.1	1.3	15.4	1.8	16.3	1.9	0.60			
20:3 n-6	0.62	0.13	0.53	0.13	0.55	0.10	0.43			
20:4 n-6	5.18	0.82	4.20	1.38	7.33	1.49	0.0033	0.39	0.032	0.0027
22:4 n-6	0.34	0.03	0.28	0.07	0.68	0.12	1.7x10^−6^	0.47	1.3x10^−5^	2.4x10^−6^
22:5 n-6	0.29	0.05	0.24	0.08	0.73	0.15	1.3x10^−6^	0.66	7.4x10^−6^	2.2x10^−6^
n-6 PUFA	22.6	2.0	20.7	3.3	25.7	3.6	0.047	0.53	0.24	0.038
n-3/n-6 PUFA ratio	0.40	0.03	0.41	0.07	0.27	0.01	0.00025	1.00	0.00061	0.00056
*Adipose tissue*										
18:3 n-3 (ALA)	1.75	0.09	1.99	0.16	1.50	0.11	5.1x10^−5^	0.012	0.012	3.4x10^−5^
20:5 n-3 (EPA)	0.13	0.01	0.15	0.02	0.03	0.003	2.0x10^−8^	0.20	2.1x10^−7^	3.5x10^−8^
22:5 n-3 (DPA)	0.28	0.02	0.32	0.03	0.13	0.01	5.5x10^−9^	0.027	1.2x10^−7^	1.2x10^−8^
22:6 n-3 (DHA)	0.46	0.04	0.51	0.06	0.12	0.01	5.0x10^−10^	0.062	1.2x10^−8^	6.7x10^−9^
n-3 PUFA	2.68	0.15	3.05	0.21	1.81	0.13	3.4x10^−8^	0.0058	2.0x10^−6^	3.2x10^−8^
18:2 n-6	15.9	0.7	17.7	1.6	15.9	1.1	0.030	0.048	1.00	0.058
20:3 n-6	0.20	0.02	0.21	0.02	0.16	0.007	0.00082	0.77	0.0035	0.0010
20:4 n-6	0.38	0.05	0.39	0.04	0.43	0.01	0.083	0.89	0.084	0.18
22:4 n-6	0.11	0.02	0.11	0.02	0.15	0.01	0.0019	1.00	0.0036	0.0039
n-6 PUFA	16.7	0.7	18.5	1.6	16.7	1.1	0.036	0.052	1.00	0.072
n-3/n-6 PUFA ratio	0.16	0.006	0.17	0.01	0.11	0.002	2.4x10^−9^	0.51	2.1x10^−8^	1.1x10^−8^

Values are mean and standard deviations, n = 5 in control group, n = 6 in baked salmon group, and n = 6 in raw salmon group.

* Baked salmon vs raw salmon.

‡ Baked salmon vs control.

† Raw salmon vs control.

(p < 0.05 were considered significant; evaluated by one-way ANOVA and Tukey HSD post-hoc test).

Contents of ALA, EPA, DPA and DHA and total amount of n-3 PUFAs were significantly higher in serum, liver and WATepi from salmon fed rats when compared to control fed rats. In skeletal muscle, EPA content was significantly higher in salmon fed rats and the concentration of ALA was higher in rats fed raw salmon compared to control. Serum levels of 18:2n-6 and 20:3n-6 were significantly higher, and that of 20:4n-6, 22:4n-6 and 22:5n-6 were significantly lower in serum from rats fed baked or raw salmon diets when compared to the control group, with no differences in serum 18:3n-6 between individual salmon groups and the control group. Salmon fed rats had significantly higher levels of 18:2n-6 and 20:3n-6, and significantly lower level of 22:5n-6 in liver when compared to controls. Salmon fed rats had significantly lower levels of 20:4n-6, 22:4n-6 and 22:5n-6 in skeletal muscle, and higher 20:3n-6 and lower 22:4n-6 in WATepi. The n-3/n-6 PUFA ratio was significantly higher in serum, liver, skeletal muscle and WATepi in rats fed salmon diets when compared to control rats.

## Discussion

Several studies in humans and animals have investigated changes in lipid metabolism after consumption of fish [29–38]. However, the preparation method is often not accounted for when interpreting the results. Differences in preparation methods of fish may explain some of the different effects on CVD risk factors associated with fish intake [5,6]. To the best of our knowledge there are no studies that directly compare the effects of baked and raw salmon intake on lipids and n-3 PUFAs in obese rats. More documentation of the health impact of differently prepared fish is needed, and the present study provides the first comparisons of preparation methods and effects on lipids and n-3 PUFA composition in serum and tissues in rats.

In the current study, the baking of salmon did not affect the fillet content of long chain n-3 PUFAs, total amino acid composition and the levels of free amino acids when compared to raw salmon fillet. We observed no differences between obese Zucker fa/fa rats fed diets containing baked or raw salmon fillet regarding growth, energy intake, lipids in serum, liver, skeletal muscle and faeces, and bile acids in serum and faeces. Baked and raw salmon intake affected n-3 PUFA composition in serum, liver and skeletal muscle of the rats in a similar manner; however, the contents of total n-3 PUFAs, ALA and DPA in WATepi were higher in rats fed raw salmon compared to rats fed baked salmon. Also the total content of n-6 PUFAs and the n-3/n-6 PUFA ratio in serum and tissues were similar between the salmon groups.

Heat treatment can increase concentrations of AGEs in fish [14] and therefore raise the AGE-burden after fish ingestion. Increased AGE-burden may induce oxidative stress, inflammation and, with time, increase the risk of CVD [12]. Similar serum concentrations of CML and MG were observed in rats fed baked or raw salmon, or control diet, suggesting that baking as a preparation method of fish does not provide enough AGEs to increase the AGE-burden in rats fed baked salmon compared to rats fed raw salmon or control diet.

Despite higher energy content in the salmon diets when compared to the control diet, the energy consumption, growth, ratio of body weight to square body length and relative organ weights were similar between all three groups. The salmon diets also contained more cholesterol compared to the control diet, but still the HDL cholesterol concentration was lower in both baked and raw salmon diet groups compared to the control group. Intake of baked salmon also resulted in lower total and LDL cholesterol concentrations, thus indicating that baked salmon affected circulating lipids to a greater extent than raw salmon feeding when compared to control. The observed effects of salmon diets on HDL cholesterol in the current study may be mediated by n-3 PUFAs, since fish oil has been shown to reduce total cholesterol in Zucker rats [39]. Also taurine, which was only detected in salmon diets, has been reported to have cholesterol lowering properties [40], and might explain differences between the salmon diets and control diet, but as the concentrations of taurine were similar in salmon diets this cannot explain why baked salmon seemed to affect circulation cholesterol to a greater extent than raw salmon. Lower ratios of lysine/arginine and methionine/glycine [41] in diets have also been suggested to explain cholesterol-reducing properties of fish protein; however, in the present study these ratios were similar in all diets. A combination of fish oil and fish protein from salmon fed to Wistar rats more efficiently lowered cholesterol concentrations than fish oil or fish protein alone [42], and we should not exclude potential synergistic effects of protein and fat from the fillets in the present study.

We have recently identified amino acid sequences with known cholesterol-lowering activity in salmon [21]. Bioactive peptides in fish can be released from proteins during digestion and food preparation [43], and the content of bioactive peptides may therefore be different in raw and heat treated fish. The more potent effect of baked salmon when compared to raw salmon on serum cholesterols in the present study might therefore be caused by a higher content of bioactive peptides with cholesterol-lowering activity in the baked salmon diet due to the heat treatment of the fillet.

In the present study we observed no difference in faecal excretion of cholesterol or bile acids between the groups, indicating that the lower concentrations of circulating HDL cholesterol in both salmon groups, and lower serum concentrations of bile acid, total and LDL cholesterols in the baked salmon group, are not a result of increased conversion of cholesterol to bile acids. These findings are in line with previous studies showing that when Zucker fa/fa rats were fed diets containing hydrolysed salmon proteins as the sole protein source, the serum concentrations of total and HDL cholesterols were lower when compared to a control group fed a casein-based diet, whereas no differences were seen in faecal cholesterol and bile acid excretion between the groups [41,44]. Lower serum bile acid concentration in rats fed baked salmon could reflect reduced activity of CYP7A1, catalysing the conversion of cholesterols to bile acids; however, the hepatic mRNA expression of CYP7A1 was not affected in Zucker fa/fa rats fed salmon hydrolysate [41].

Intake of diets containing baked or raw salmon fillet did not affect hepatic cholesterol levels when compared to the control diet in the present study. This is in contrast to a previous study, where we observed larger accumulation of cholesterol in liver together with lower circulating concentrations of total and HDL cholesterols when Wistar rats consumed salmon oil and salmon protein hydrolysate in combination [42]. Diets containing salmon oil and salmon protein hydrolysate either alone or in combination did not affect the hepatic HMG-CoA reductase activity in Wistar rats, suggesting that cholesterol synthesis in liver was not changed [42]; however, hepatic HMG-CoA reductase activity was induced when Zucker fa/fa rats were fed a salmon protein hydrolysate-containing diet [44]. Others report higher activity of hepatic microsomal HMG-CoA reductase in Wistar rats fed EPA ethyl ester and lower activity of the same enzyme in these rats fed DHA ethyl esters [45]. Discrepancy between findings in different rat studies may be caused by dissimilar design, e.g. different rat strain, different doses of protein/fat or different types of fish proteins. Thus, the lower concentrations of serum total, HDL and LDL cholesterols in rats fed baked salmon, and the lower HDL cholesterol in the raw salmon group, may be due to reduced biosynthesis of cholesterol in rats fed salmon diets, but other mechanisms responsible for these findings should not be ruled out.

Reduction in circulating TAG is considered the most consistent effect of n-3 PUFA consumption [46]. In the present study, serum TAG were significantly higher in rats fed baked salmon (p = 0.015) and tended to be higher (p = 0.067) in rats fed raw salmon compared to control fed rats, with no differences in hepatic TAG levels between the groups. Previously, we have observed no effects on serum TAG with diets containing 25% of protein from cod or salmon [20,21] and higher serum TAG with diet containing 25% of protein from herring [21] in Zucker fa/fa rats. This indicates that consumption of different types of fish protein as well as fish fillet may affect serum TAG in obese rats. Circulating TAG is mainly present in chylomicrons or VLDL particles and whereas chylomicrons are most abundant in the fed state, VLDL particles are released from liver in the fasting state. Since serum samples in the current study were obtained after a 12 h overnight fast, circulating TAG would normally be packed in VLDL particles, reflecting elevated secretion of TAG from the liver. However, Zucker fa/fa rats have an attenuated clearance of chylomicrons [47] and as salmon diets had higher fat content, a 12 h fast might not be sufficient for clearance of chylomicrons from the circulation. A slow clearance of chylomicrons may explain the higher serum TAG in the rats fed baked salmon when compared to controls.

The histological examination of the hepatic lipid droplets, mainly consisting of TAG and cholesteryl esters, suggests that the storage of neutral fat may be influenced by salmon-containing diets, in particular by raw salmon intake. We found significantly lower lipid content in liver from rats fed the raw salmon diet, and a weak tendency to lower hepatic lipid content in rats fed baked salmon, when compared to the control group. The Zucker fa/fa rat develops fatty liver [19], and a possible beneficial effect of fatty fish intake on this condition should be explored further.

Suboptimal vitamin D status has been associated with CVD [48]. The AIN93G diet, which the feed in the present study is based on, is designed for optimal animal health and is supplemented with 25 µg vitamin D3/kg feed. In the present study rats were fed salmon fillet with low vitamin D3 concentrations providing only 8 µg additional vitamin D3 per kg feed. Rats fed diets containing baked or raw salmon had similar serum concentrations of vitamin D as the control group, indicating that the additional vitamin D3 provided by salmon was not sufficient to affect the vitamin D status further in the present study.

Concentrations of ALA, EPA, DPA, DHA and total n-3 PUFAs in serum, liver and skeletal muscle were similar between rats fed baked or raw salmon diets. Rats fed raw salmon had higher concentrations of ALA, DPA and total n-3 PUFAs in WATepi compared to rats fed baked salmon, thus it appears that n-3 fatty acids were more efficiently incorporated in WATepi when rats were fed raw salmon as compared to baked salmon. Although significant, the differences in amount of ALA and DPA in WATepi are relatively small and the clinical relevance of this finding is uncertain. Both salmon diets led to higher total n-3 PUFA content in serum, liver and WATepi compared to control, and a higher n-3/n-6 PUFA ratio in serum, liver, skeletal muscle and WATepi, which could be expected due to the higher n-3 PUFA content of salmon diets.

The present study provides a comparison of amino acids and n-3 PUFAs in baked and raw salmon fillets, and compares the effects of baked and raw salmon consumption as part of a balanced AIN-93G diet in rats. To conclude, baking as a preparation method does not seem to alter the protein and fat qualities of salmon fillets, and intake of baked and raw salmon fillets provided similar effects on lipids and n-3 fatty acid composition in serum and tissues in Zucker fa/fa rats.

## References

[1] Hu FB, Bronner L, Willett WC, et al Fish and omega-3 fatty acid intake and risk of coronary heart disease in women. Jama. 2002;287:1815–1821 ​​​​​ 11939867 10.1001/jama.287.14.1815

[2] Albert CM, Hennekens CH, O’Donnell CJ, et al Fish consumption and risk of sudden cardiac death. Jama. 1998;279:23–28. 9424039 10.1001/jama.279.1.23

[3] Morris MC, Manson JE, Rosner B, et al Fish consumption and cardiovascular disease in the physicians’ health study: a prospective study. Am J Epidemiol. 1995;142:166–175. 7598116 10.1093/oxfordjournals.aje.a117615

[4] Harris WS. Are n-3 fatty acids still cardioprotective? Curr Opin Clin Nutr Metab Care. 2013;16:141–11. 23196817 10.1097/MCO.0b013e32835bf380

[5] Mozaffarian D, Lemaitre RN, Kuller LH, et al Cardiac benefits of fish consumption may depend on the type of fish meal consumed: the Cardiovascular Health Study. Circulation. 2003;107:1372–1377. 12642356 10.1161/01.cir.0000055315.79177.16

[6] Mozaffarian D, Psaty BM, Rimm EB, et al Fish intake and risk of incident atrial fibrillation. Circulation. 2004;110:368–373. 15262826 10.1161/01.CIR.0000138154.00779.A5 PMC1201400

[7] Bowen KJ, Harris WS, Kris-Etherton PM Omega-3 fatty acids and cardiovascular disease: are there benefits? Curr Treat Options Cardiovasc Med. 2016;18:69. 27747477 10.1007/s11936-016-0487-1 PMC5067287

[8] Chiesa G, Busnelli M, Manzini S, et al Nutraceuticals and bioactive components from fish for dyslipidemia and cardiovascular risk reduction. Mar Drugs. 2016;14:113. 10.3390/md14060113 PMC4926072 27338419

[9] Castro-González I, Maafs-Rodríguez AG, Pérez-Gil Romo F Effect of six different cooking techniques in the nutritional composition of two fish species previously selected as optimal for renal patient’s diet. J Food Sci Technol. 2015;52:4196–4205. 26139884 10.1007/s13197-014-1474-8 PMC4486557

[10] Candela M, Astiasarán I, Bello J Deep-fat frying modifies high-fat fish lipid fraction. Journal of Agricultural and Food Chemistry. 1998;46:2793–2796.

[11] Echarte M, Zulet MA, Astiasaran I Oxidation process affecting fatty acids and cholesterol in fried and roasted salmon. J Agric Food Chem. 2001;49:5662–5667. 11714374 10.1021/jf010199e

[12] Uribarri J, del Castillo MD, de la Maza MP, et al Dietary advanced glycation end products and their role in health and disease. Adv Nutr. 2015;6:461–473. 26178030 10.3945/an.115.008433 PMC4496742

[13] Goldberg T, Cai W, Peppa M, et al Advanced glycoxidation end products in commonly consumed foods. J Am Diet Assoc. 2004;104:1287–1291. 15281050 10.1016/j.jada.2004.05.214

[14] Uribarri J, Woodruff S, Goodman S, et al Advanced glycation end products in foods and a practical guide to their reduction in the diet. J Am Diet Assoc. 2010;110:911–916. 20497781 10.1016/j.jada.2010.03.018 PMC3704564

[15] Clarke RE, Dordevic AL, Tan SM, et al Dietary advanced glycation end products and risk factors for chronic disease: a systematic review of randomised controlled trials. Nutrients. 2016;8:125. 26938557 10.3390/nu8030125 PMC4808855

[16] Rohrmann S, Linseisen J, Becker N, et al Cooking of meat and fish in Europe–results from the European Prospective Investigation into Cancer and Nutrition (EPIC). Eur J Clin Nutr. 2002;56:1216–1230 ​​​​​ 12494307 10.1038/sj.ejcn.1601494

[17] Altintzoglou T, Heide M, Wien AH, et al Traditional sushi for modern consumers: a comparison between sushi consumption behavior in Japan and Norway. Journal of Food Products Marketing. 2016;22:717–732.

[18] Edwards PA Global sushi: eating and identity. Perspectives on Global Development and Technology. 2012;11:211–225.

[19] de Artinano AA, Castro MM Experimental rat models to study the metabolic syndrome. Br J Nutr. 2009;102:1246–1253. 19631025 10.1017/S0007114509990729

[20] Drotningsvik A, Mjøs SA, Høgøy I, et al A low dietary intake of cod protein is sufficient to increase growth, improve serum and tissue fatty acid compositions, and lower serum postprandial glucose and fasting non-esterified fatty acid concentrations in obese Zucker fa/fa rats. Eur J Nutr. 2015;54:1151–1160. 25380663 10.1007/s00394-014-0793-x

[21] Drotningsvik A, Mjos SA, Pampanin DM, et al Dietary fish protein hydrolysates containing bioactive motifs affect serum and adipose tissue fatty acid compositions, serum lipids, postprandial glucose regulation and growth in obese Zucker fa/fa rats. Br J Nutr. 2016;116:1336–1345. 27751188 10.1017/S0007114516003548

[22] Reeves PG, Nielsen FH, Fahey GC Jr. AIN-93 purified diets for laboratory rodents: final report of the American Institute of Nutrition ad hoc writing committee on the reformulation of the AIN-76A rodent diet. J Nutr. 1993;123:1939–1951. 8229312 10.1093/jn/123.11.1939

[23] Sissener NH, Waagbø R, Rosenlund G, et al Reduced n-3 long chain fatty acid levels in feed for Atlantic salmon (Salmo salar L.) do not reduce growth, robustness or product quality through an entire full scale commercial production cycle in seawater. Aquaculture. 2016;464:236–245.

[24] Bligh EG, Dyer WJ A rapid method of total lipid extraction and purification. Canadian Journal of Biochemistry and Physiology. 1959;37:911–917. 13671378 10.1139/o59-099

[25] Suckling KE, Benson GM, Bond B, et al Cholesterol lowering and bile acid excretion in the hamster with cholestyramine treatment. Atherosclerosis. 1991;89:183–190. 1793446 10.1016/0021-9150(91)90059-c

[26] Meier S, Mjøs SA, Joensen H, et al Validation of a one-step extraction/methylation method for determination of fatty acids and cholesterol in marine tissues. J Chromatogr A. 2006;1104:291–298. 16343517 10.1016/j.chroma.2005.11.045

[27] Sciotto C, Mjos SA Trans isomers of EPA and DHA in omega-3 products on the European market. Lipids. 2012;47:659–667. 22566205 10.1007/s11745-012-3672-3

[28] Wasta Z, Mjøs SA A database of chromatographic properties and mass spectra of fatty acid methyl esters from omega-3 products. J Chromatogr A. 2013;1299:94–102. 23773584 10.1016/j.chroma.2013.05.056

[29] Telle-Hansen VH, Larsen LN, Hostmark AT, et al Daily intake of cod or salmon for 2 weeks decreases the 18: 1n-9/18:0ratio and serum triacylglycerols in healthy subjects. Lipids. 2012;47:151–160. 22139893 10.1007/s11745-011-3637-y

[30] Zhang J, Wang C, Li L, et al Inclusion of Atlantic salmon in the Chinese diet reduces cardiovascular disease risk markers in dyslipidemic adult men. Nutr Res. 2010;30:447–454. 20797476 10.1016/j.nutres.2010.06.010

[31] Ouellet V, Weisnagel SJ, Marois J, et al Dietary cod protein reduces plasma C-reactive protein in insulin-resistant men and women. J Nutr. 2008;138:2386–2391. 19022962 10.3945/jn.108.092346

[32] Lindqvist H, Sandberg AS, Undeland I, et al Influence of herring (Clupea harengus) and herring fractions on metabolic status in rats fed a high energy diet. Acta Physiol (Oxf). 2009;196:303–314. 19076113 10.1111/j.1748-1716.2008.01948.x

[33] Hagen IV, Helland A, Bratlie M, et al High intake of fatty fish, but not of lean fish, affects serum concentrations of TAG and HDL-cholesterol in healthy, normal-weight adults: a randomised trial. Br J Nutr. 2016;116:648. 27363518 10.1017/S0007114516002555

[34] Lara JJ, Economou M, Wallace AM, et al Benefits of salmon eating on traditional and novel vascular risk factors in young, non-obese healthy subjects. Atherosclerosis. 2007;193:213–221. 17069820 10.1016/j.atherosclerosis.2006.06.018

[35] Gunnarsdottir I, Tomasson H, Kiely M, et al Inclusion of fish or fish oil in weight-loss diets for young adults: effects on blood lipids. Int J Obes (Lond). 2008;32:1105–1112. 18490931 10.1038/ijo.2008.64

[36] Mori TA, Bao DQ, Burke V, et al Dietary fish as a major component of a weight-loss diet: effect on serum lipids, glucose, and insulin metabolism in overweight hypertensive subjects. Am J Clin Nutr. 1999;70:817–825. 10539741 10.1093/ajcn/70.5.817

[37] Zhang X, Beynen AC Influence of dietary fish proteins on plasma and liver cholesterol concentrations in rats. Br J Nutr. 1993;69:767–777. 8329352 10.1079/bjn19930077

[38] Shukla A, Bettzieche A, Hirche F, et al Dietary fish protein alters blood lipid concentrations and hepatic genes involved in cholesterol homeostasis in the rat model. Br J Nutr. 2006;96:674–682. 17010226

[39] Mohan PF, Phillips FC, Cleary MP Metabolic effects of coconut, safflower, or menhaden oil feeding in lean and obese Zucker rats. Br J Nutr. 1991;66:285–299. 1760446 10.1079/bjn19910032

[40] Chen W, Guo JX, Chang P The effect of taurine on cholesterol metabolism. Mol Nutr Food Res. 2012;56:681–690. 22648615 10.1002/mnfr.201100799

[41] Gudbrandsen OA, Wergedahl H, Liaset B, et al Dietary proteins with high isoflavone content or low methionine-glycine and lysine-arginine ratios are hypocholesterolaemic and lower the plasma homocysteine level in male Zucker fa/fa rats. Br J Nutr. 2005;94:321–330. 16176601 10.1079/bjn20051496

[42] Wergedahl H, Gudbrandsen OA, Røst TH, et al Combination of fish oil and fish protein hydrolysate reduces the plasma cholesterol level with a concurrent increase in hepatic cholesterol level in high-fat-fed Wistar rats. Nutrition. 2009;25:98–104. 18752928 10.1016/j.nut.2008.07.005

[43] Moller NP, Scholz-Ahrens KE, Roos N, et al Bioactive peptides and proteins from foods: indication for health effects. Eur J Nutr. 2008;47:171–182. 18506385 10.1007/s00394-008-0710-2

[44] Wergedahl H, Liaset B, Gudbrandsen OA, et al Fish protein hydrolysate reduces plasma total cholesterol, increases the proportion of HDL cholesterol, and lowers Acyl-CoA:cholesterol acyltransferase activity in liver of zucker rats. J Nutr. 2004;134:1320–1327. 15173391 10.1093/jn/134.6.1320

[45] Frøyland L, Vaagenes H, Asiedu DK, et al Chronic administration of eicosapentaenoic acid and docosahexaenoic acid as ethyl esters reduced plasma cholesterol and changed the fatty acid composition in rat blood and organs. Lipids. 1996;31:169–178. 8835405 10.1007/BF02522617

[46] Balk EM, Lichtenstein AH, Chung M, et al Effects of omega-3 fatty acids on serum markers of cardiovascular disease risk: a systematic review. Atherosclerosis. 2006;189:19–30. 16530201 10.1016/j.atherosclerosis.2006.02.012

[47] Redgrave TG Catabolism of chylomicron triacylglycerol and cholesteryl ester in genetically obese rats. J Lipid Res. 1977;18:604–612. 903708

[48] Manousopoulou A, Al-Daghri NM, Garbis SD, et al Vitamin D and cardiovascular risk among adults with obesity: a systematic review and meta-analysis. Eur J Clin Invest. 2015;45:1113–1126. 26222607 10.1111/eci.12510

